# Long-distance transport of radioactive plume by nocturnal local winds

**DOI:** 10.1038/srep36584

**Published:** 2016-11-16

**Authors:** Takao Yoshikane, Kei Yoshimura, Eun-Chul Chang, Akane Saya, Taikan Oki

**Affiliations:** 1 Institute of Industrial Science, The University of Tokyo, 4-6-1, Komaba, Meguro-ku, Tokyo, 153-8505, Japan; 2 Atmosphere and Ocean Research Institute, The University of Tokyo, 5-1-5, Kashiwanoha, Kashiwa-shi, Chiba, 277-8564, Japan; 3 Department of Atmospheric Science, Kongju National University, 56 Gongjudaehak-ro, Gongju-si, Chungcheongnam-do, 32588, Korea

## Abstract

Radioactive plumes can spread far and wide depending on wind conditions. The plumes often frequently reached the Tokyo metropolitan area, which is approximately 200 km away from the Fukushima Daiichi nuclear power plant, under spatially heterogeneous wind fields in March 2011. To reduce exposure to radioactive plumes, the behaviour of the plumes must be known. However, the transport mechanism of radioactive plumes is not fully understood. Using a regional climate model, we show that multiple diurnal cycle processes play a key role in the frequent transport of radioactive plumes to the Tokyo metropolitan area. The observed data and hindcast results indicate that the radioactive plume moves along the local winds, which comprise the northeasterly local wind (NELW) associated with the meso-scale low-pressure system (meso-low) and the northerly sea wind (NSW) during the night. The long-term analysis and sensitivity simulations also show the nocturnal processes that the NELW caused by the meso-low and the NSW are formed east of the Tokyo metropolitan area and from Fukushima offshore east of the Tokyo metropolitan area, respectively, when neither winter monsoons nor extra-tropical cyclones are predominant. These findings indicate that the radioactive plumes could reach faraway places frequently via nocturnal local processes.

Radioactive plumes can scatter widely under the strong influence of the weather^1^,^2^,^3^,^4^,^5^,^6^,^7^,^8^. After the accident at the Fukushima Daiichi nuclear power plant in March 2011, the Japanese government evacuated the area within a 20-km radius of the power plant and advised residents within a 20-km to 30-km radius of the power plant to stay inside their homes^9^. However, high air doses were observed in faraway places outside the 30-km radius (Fig. 1a,b). In such situations, exposure should be minimized because the released radioactive material (^131^I) is assumed to have the potential to cause thyroid cancer^10^. Therefore, when and where radioactive plumes will travel should be known in advance.

The movement of a radioactive plume is not only influenced by large-scale events, such as monsoons and extra-tropical cyclones, but also by local-scale events^4^,^5^. For example, local-scale events, including land/sea breezes, are predominant under calm weather conditions^11^,^12^. A land/sea breeze can cause severe atmospheric pollution even in areas that are distant from the emission source^13^,^14^,^15^. A contamination could occur in a specific area because of the local circulation if large amounts of radioactive materials are emitted over a long period.

A large quantity of radioactive ^131^I, estimated to be between 1.8 × 10^12^ and 8.9 × 10^15^ Bq h^−1^, was released from the Fukushima Daiichi nuclear power plant by the end of March 2011^16^,^17^,^18^. To represent the transport and deposition distribution of radioactive materials in Japan, several numerical simulations have been performed using the estimated emission data^3^,^4^,^5^,^6^,^7^,^8^,^16^,^17^,^18^. However, simulating the distributions is difficult because many uncertainties affect numerical simulations. One such uncertainty is the chaotic behaviour of the atmosphere^19^,^20^, which amplifies prediction errors resulting from imperfections in the model formulation or the sensitive dependence on the initial conditions. Indeed, if chaotic behaviour were predominant, the movement of the radioactive plume would be difficult to predict accurately.

In contrast, predicting the movement of a radioactive plume would be relatively simple if large-scale events, such as monsoons and extra-cyclones, were predominant because the wind field would be expected to be temporally constant and spatially homogeneous. However, radioactive plumes often reached the Tokyo metropolitan area, even under spatially heterogeneous wind fields^5^. The types of atmospheric events that might have affected the wind field and the mechanisms by which the radioactive plumes travelled over long distances remain poorly understood. The chaotic behaviour of the atmosphere might be associated with the movement of the radioactive plume. Here, we investigate the mechanism of radioactive plume transport from Fukushima to the Tokyo metropolitan area using a regional climate model.

## Results

**Figure 1:**
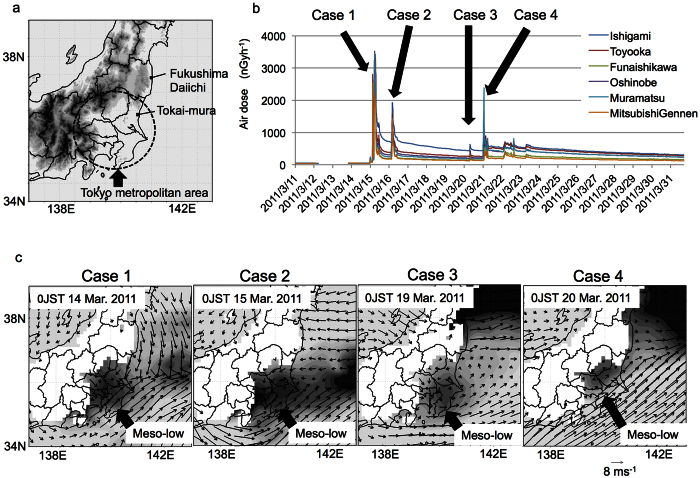
A common feature of the atmospheric fields when a high air dose was observed in the Tokyo metropolitan area. (**a**) The locations of Fukushima, Tokai-mura, and the Tokyo metropolitan area. (**b**) Time variations of the observed air doses at the observation sites in Tokai-mura. Cases 1, 2, 3, and 4 correspond to the spikes in the air dose. (**c**) The wind field and geo-potential height of MSM-GPV (975 hPa) at midnight before each of the four cases. Dark areas indicate low pressure. The maps were created by using GrADS 2.0.1 (http://cola.gmu.edu/grads/) (**a,c**) and Microsoft Excel for Mac 2011 (**b**).

High air doses, indicated by the spike in Fig. 1b, were often observed at Tokai-mura in the eastern coastal region of the Tokyo metropolitan area in the morning. At approximately the same time, the NSW and the NELW commonly occurred near the coastal area of the northeastern region of the Tokyo metropolitan area at 975 hPa (see Supplementary Fig. S1), whereas these winds were not detected at 850 hPa (see Supplementary Fig. S2). Another common feature, the nocturnal meso-low, formed in the Tokyo metropolitan area before the high dose rates were observed east of the Tokyo metropolitan area in the morning (Fig. 1c). We conducted a hindcast (HC run) to confirm the relationship between the observed high air radiation doses and the radioactive plume simulated using a regional climate model^21^ (see Methods). The simulated radioactive plume occurred from Fukushima to the northeastern part of the Tokyo metropolitan area in all cases (see Supplementary Fig. S3).

We assumed that the NSW, NELW, and nocturnal meso-low strongly influenced the radioactive plume transport when neither winter monsoons nor extra-cyclones were predominant. Some diurnal cycle processes could have formed the NSW, NELW, and nocturnal meso-low if the diurnal variations of those atmospheric fields were confirmed in the long-term composite data during calm weather. To verify this hypothesis, we defined a typical day when diurnal wind was observed as a calm day using station data for the central part of the Tokyo metropolitan area. The four cases shown in Fig. 1 were included in the calm day. The diurnal variations were investigated by using the operational meteorological analysis dataset for March from 2008 to 2014 (see Supplementary Fig. S4 and Methods). Seven-year composite would be sufficient to detect the signal of diurnal cycle significantly.

**Figure 2:**
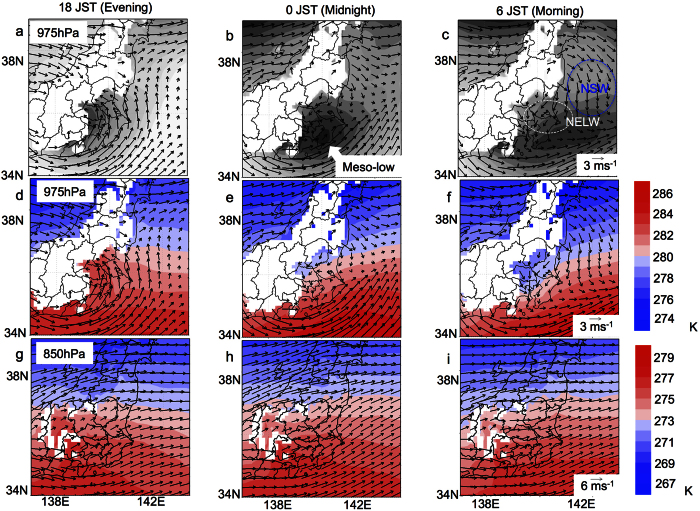
Diurnal variation of the wind fields under calm conditions. Diurnal variation of the composite data of wind fields, geo-potential height, and temperature at 975 hPa and 850 hPa on calm days from 2008 to 2014 according to the MSM-GPV data. The dark areas indicate areas of low geo-potential height (low pressure). The maps were created by using GrADS 2.0.1 (http://cola.gmu.edu/grads/).

The results demonstrated that the NSW, NELW, and meso-low were clearly evident in the composite of the calm day (Fig. 2b,c) at 975 hPa at night, whereas these atmospheric fields were unclear at 850 hPa (Fig. 2g–i). The meso-low could strongly influence the formation of the NELW. Additionally, the NSW and NELW could be formed as gravity currents induced by the meridional temperature gradient because no predominant forcing exists except for the temperature gradient at night under calm conditions. In contrast, the onshore wind, which is intensified by the heat-low at the mountains of central Japan^14^, is clearly evident in the daytime (Fig. 2a,d). Almost 30% of the days in March from 2008 to 2014 were calm days (see Supplementary Fig. S5). Thus, diurnal cycle processes are not rare events but are important contributors to the regional climate in March.

The nocturnal meso-low forms in various areas worldwide^22^,^23^,^24^,^25^,^26^. The topographical heat-low in the daytime could be a trigger of the meso-low^23^. However, the nocturnal meso-low has been observed to persist until the morning (Fig. 2c). If the convergence caused by the NSW sustains the meso-low, the topographic effect and meridional temperature gradient could be important in the formation of the meso-low.

**Figure 3:**
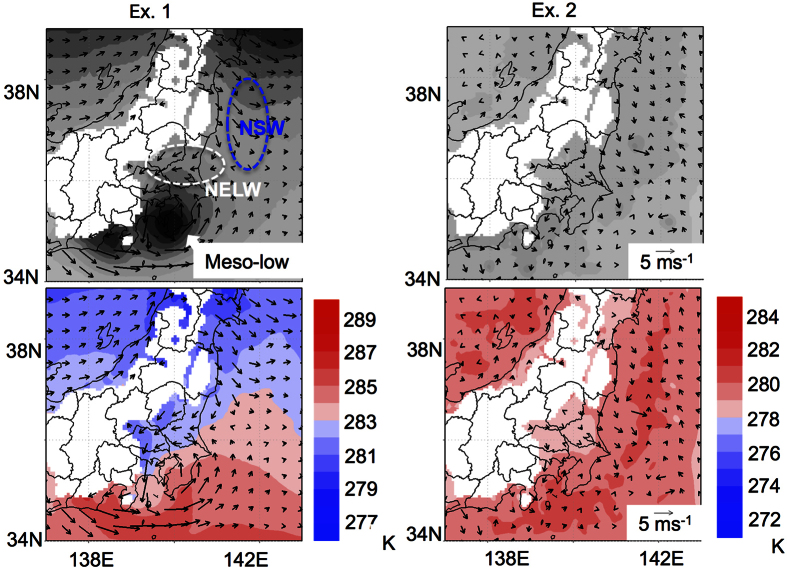
Sensitivity test. The wind fields, geo-potential heights, and temperatures at 975 hPa in the morning (6 JST) of Ex. 1 and Ex. 2. The atmospheric fields of the global zonal mean and area-averaged values in March 2011 were applied as the lateral boundary conditions of Ex. 1 and Ex. 2, respectively. The maps were created by using GrADS 2.0.1 (http://cola.gmu.edu/grads/).

To elucidate the formation mechanisms of the NSW, NELW, and meso-low, we conducted simple sensitivity tests (see Methods). The effect of the meridional temperature gradient was investigated by adapting a monthly averaged global zonal mean field in March 2011 as the initial and boundary conditions (Ex. 1); the effect of geography, including the land/sea contrast, was investigated by adapting the area-averaged atmospheric field around east Japan (Ex. 2) (see Supplementary Fig. S6). The result shows that Ex. 1 simulates the NSW, NELW, and meso-low but Ex. 2 does not (Fig. 3). This finding indicates that the meridional temperature gradient is essential in the formation of the diurnal cycle of the atmospheric field.

## Discussion

**Figure 4:**
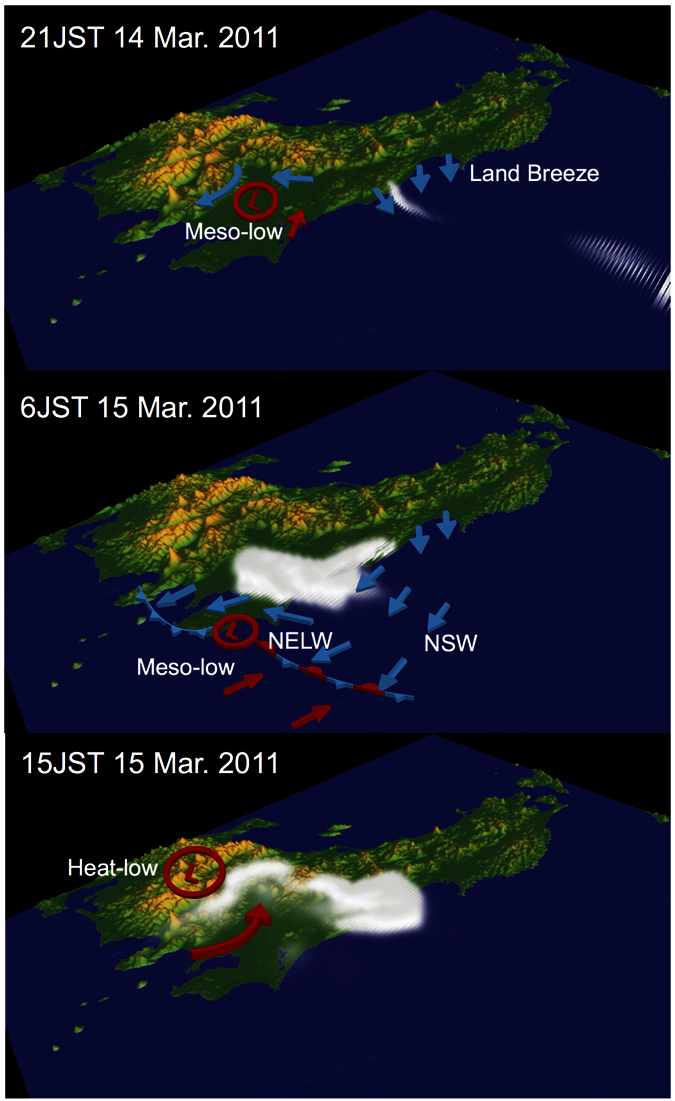
Long-distance transport of the radioactive plume via multiple diurnal processes. The 3D image of the mixing ratio of ^131^I in Case 1. The maps were created by using Volume Data Visualizer for Google Earth (VDVGE) 1.1.7 ESC JAMSTEC (https://www.jamstec.go.jp/esc/research/Perception/vdvge.ja.html) and Adobe Illustrator CS5 (http://www.adobe.com).

A schematic of the transport of radioactive materials is presented in Fig. 4. The radioactive materials are transported to an area offshore of Fukushima by the land breeze, and then, the plume moves to the south via the NSW (Fig. 4a). In the morning, the radioactive plume flows into the Tokyo metropolitan area via the NELW, which is formed by the nocturnal meso-low (Fig. 4b). In the afternoon, the plume moves to the mountain area located to the northeast of the Tokyo metropolitan area because of the intensified sea breeze induced by the heat-low over the mountains in central Japan (Fig. 4c).

The northeasterly wind accompanied by rain is often observed around the Tokyo metropolitan area during winter mornings^27^,^28^,^29^. The developed nocturnal meso-low is responsible for this precipitation. Consequently, it was reassuring that no rainfall was detected on 15 March 2011, when the highest air doses were observed (case 1 in Fig. 1). If rainfall had occurred, the serious contamination would have also caused in the Tokyo metropolitan area.

In the seven-year simulation with the constant emission of radioactive materials (CE run), a high deposition of ^131^I was simulated from Fukushima to the Tokyo metropolitan area in the morning, with increased deposition occurring the mountains located east of the Tokyo metropolitan area in the evening (see Supplementary Fig. S7 and Methods). The diurnal variation of the deposition could be explained by the movement of the radioactive plume corresponding to the diurnal wind field shown in Fig. 4. Thus, diurnal processes strongly influence the deposition distribution.

The amounts of radioactive materials deposited, especially ^137^Cs, depend strongly on the precipitation^30^. Generally, precipitation is difficult to simulate using a numerical model quantitatively with high accuracy because of the non-linearity of the precipitation process. Therefore, accurately estimating the deposition at a specific point without observations would be difficult. Therefore, using only the simulated deposition (exposure by groundshine), determining whether immediate evacuation should be enforced is problematic. Our new findings will be useful for determining the time to take shelter to avoid exposure to the radioactive plume (by cloudshine and/or intake) when a large-scale event is not predominant. Additionally, by applying the transport mechanism clarified here, we could potentially reduce the uncertainties relating to the deposition of radioactive materials. Therefore, we should continue improving existing numerical models to more accurately represent the local circulation caused by diurnal cycle processes. This finding could also useful to improve the local depositions simulated by a global circulation model^31^.

Generally, local circulation is not simple because various factors, such as land use, geographical features, and synoptic wind, strongly influence the local wind field^12^. The findings of this study indicate that when a severe nuclear power plant accident occurs, radioactive plumes could reach faraway places via multiple diurnal cycle processes. Therefore, establishing a detailed mechanism of local circulation in every area is necessary to make any progress in reducing the uncertainties related to exposure.

## Methods

### HC simulation

We conducted the HC run from 11 to 31 March 2011 to simulate the distribution of the radioactive plume during the period in which high air doses were observed in Tokai-mura using the Isotopic Regional Spectral Model (IsoRSM)^21^. The emission height conveniently set to the surface because the emission height was estimated to range from 20 to 120 m (fairly near the surface) by the reverse-estimation method^16^,^17^,^18^. We also used the time variation data of the release rate estimated by the reverse-estimation method^18^. The meso-scale model grid point value (MSM-GPV) datasets, which were provided by the Japan Meteorological Agency, served as the initial and lateral boundary conditions of the model. The spectral nudging method was applied to the lateral boundary data. The calculation domain is shown in Supplementary Fig. S8. The grid spacing was 5 km in this domain. A semi-Lagrangian model was used to calculate the transport of radioactive materials^32^. The deposition distributions of ^137^Cs are reasonably simulated around the Fukushima-Daiichi nuclear power plant and around the Tokyo metropolitan area. (see Supplementary Fig. S9). The large part of the simulated depositions is within the range from 0.1 to 10 times of the observed depositions (see Supplementary Fig. S10). The timings of the deposition of ^131^I and ^137^Cs are also reasonably simulated as shown in Supplementary Figs S11 and S12. The Gaussian plume model often used to investigate the dispersion of the radioactive materials^33^. However the model is limited in the application to the plain area and the horizontally homogeneous wind field. Actually, the plain area is very small and wind field is not always homogeneous. Therefore, we used the regional atmospheric model with semi-Lagrangian transport model to clarify the transport mechanism from Fukushima to Tokyo metropolitan area.

### Long-term analysis

As shown in Supplementary Fig. S4, the diurnal cycle was clearly reflected by the time variation of the area-averaged temperature and meridional wind in the central part of the Tokyo metropolitan area. In contrast, the diurnal cycle was unclear when monsoons or extra-tropical cyclones were predominant, as shown from 16 to 18 March and from 21 to 23 March. Based on these features, we defined a calm day as follows. First, the area-averaged daily mean wind speed was less than 1.2 m/s. Second, the daily maximum and minimum of the area-averaged meridional winds were positive and negative, respectively. The area-averaged values were calculated using the 36 data points from the station data of the Automated Meteorological Data Acquisition System (AMeDAS) in the region from 35N to 36N and from 139E to 141E. A daily mean was calculated using the data from 0UTC (9JST) to 23UTC (8JST). Four cases in Fig. 1b satisfy those conditions.

### Sensitivity tests

The monthly averaged global zonal mean values of the horizontal and meridional winds, temperature, geo-potential height, and relative humidity of the NCEP reanalysis data^34^ and sea surface temperature of the OISST data^35^ in March 2011 were used as the initial and boundary data in Ex. 1; the monthly averaged regional mean data except for winds from 30N to 40N and from 135E to 145E of the NCEP reanalysis data and the OISST data were used in Ex. 2. The zonal and meridional winds are set to 0.1 ms^-1^ in order to show the effect of Land/Sea contrast clearly. The simulation started at 0 UTC on 15 March and ended at 21 UTC on 16 March.

### Long-term simulation by constant emission

We conducted the seven-year simulation with a fixed release rate in March from 2008 to 2014 (CE run) to investigate the diurnal change in the deposition distribution (see Supplementary Table S1). The wet deposition (washout process) was calculated by:


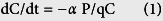


where C is atmospheric concentration of radioactive materials, α is the washout coefficient (0.5), and P and q are the vertically integrated water condensation and the water vapour in the atmospheric column, respectively^36^.

The dry deposition was calculated by:


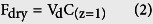


where V_d_ is the deposition speed, and C_(z=1)_ is the concentration in the lowest layer. The V_d_ values of ^137^Cs and ^131^I are 1 × 10^−3^ ms^−1^ and 5 × 10^−3^ ms^−1^ over the sea and 5 × 10^−3^ ms^−1^ and 2.5 × 10^−2^ ms^−1^ over the land, respectively^37^. The other calculation conditions were the same as in the HC run.

## Additional Information

**How to cite this article**: Yoshikane, T. *et al.* Long-distance transport of radioactive plume by nocturnal local winds. *Sci. Rep.* **6**, 36584; doi: 10.1038/srep36584 (2016).

**Publisher’s note:** Springer Nature remains neutral with regard to jurisdictional claims in published maps and institutional affiliations.

## Supplementary Material

Supplementary Information
