# Supplementary material for: Long-distance transport of radioactive plume by nocturnal local winds
Source: Sci Rep. 2016 Nov 16;6:36584. doi: 10.1038/srep36584 (PMC5111080; doi:10.1038/srep36584)
Supplement: Supplementary Information [file srep36584-s1.pdf]

Supplementary information

Long-distance transport of radioactive plume by nocturnal local winds

Takao Yoshikane[1], Kei Yoshimura[1], Eun-Chul Chang[2], Akane Saya[1], & Taikan Oki[1]

[1]Institute of Industrial Science, University of Tokyo, 4-6-1, Komaba, Meguro-ku, Tokyo, 153-8505, Japan

[2]Department of Atmospheric Science, Kongju National University. #446 College of Natural Science, 56 Gongjudaehak-ro, Gongju-si, Chungcheongnam-do, 314-701, Korea

Correspondence to T. Yoshikane (takao-y@iis.u-tokyo.ac.jp)

Run types	Nudging	Simulation term	Boundary condition	Radioactive materials release
HC	ON	11 ~ 31 Mar. 2011	MSM-GPV (6-hourly data)	Estimated values by Kobayashi et al. (2013)
Ex. 1	OFF	2 days Solar time(0UTC 15~16 Mar.)	Global zonal mean and monthly averaged data of Mar. 2011<br>(NCEP reanalysis data)	No release
Ex. 2	OFF	2 days Solar time(0UTC 15~16 Mar.)	Global zonal mean latitudinal average from 32.5N to 40N of Mar. 2011<br>(NCEP reanalysis)<br>SST: 285.36K (OISST)	No release
CE	ON	1 ~ 31 Mar. from 2008 to 2014	MSM-GPV (6-hourly data)	Maximum estimated values by Terada et al. (2012) during the time-integration constantly

Supplementary Table S1: **Detailed simulation design.** The map was created by using Microsoft Power Point for Mac 2011.

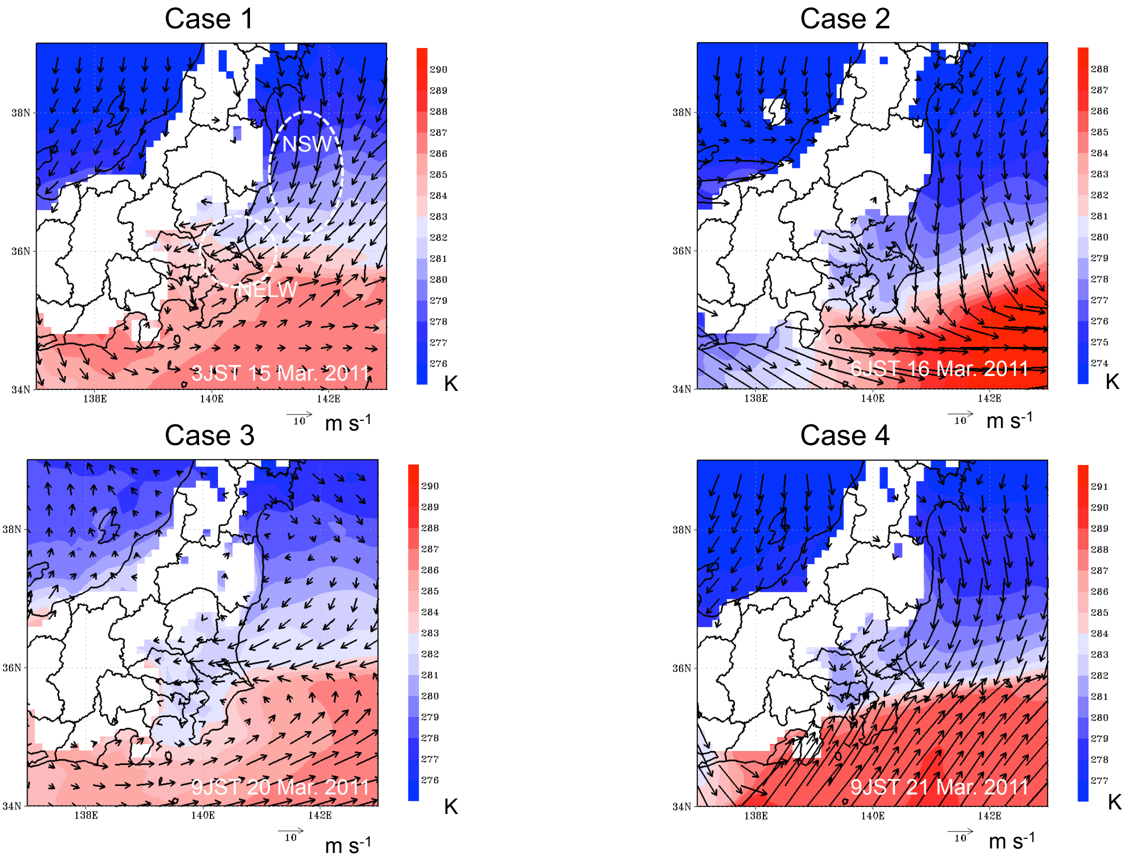

Supplementary Figure S1: **Near surface airflows around Tokyo metropolitan area.**

The wind and the temperature fields at 975hPa (MSM-GPV) in the morning when the high air dose observed in Fig.1b. The maps were created by using GrADS 2.0.1 (http://cola.gmu.edu/grads/).

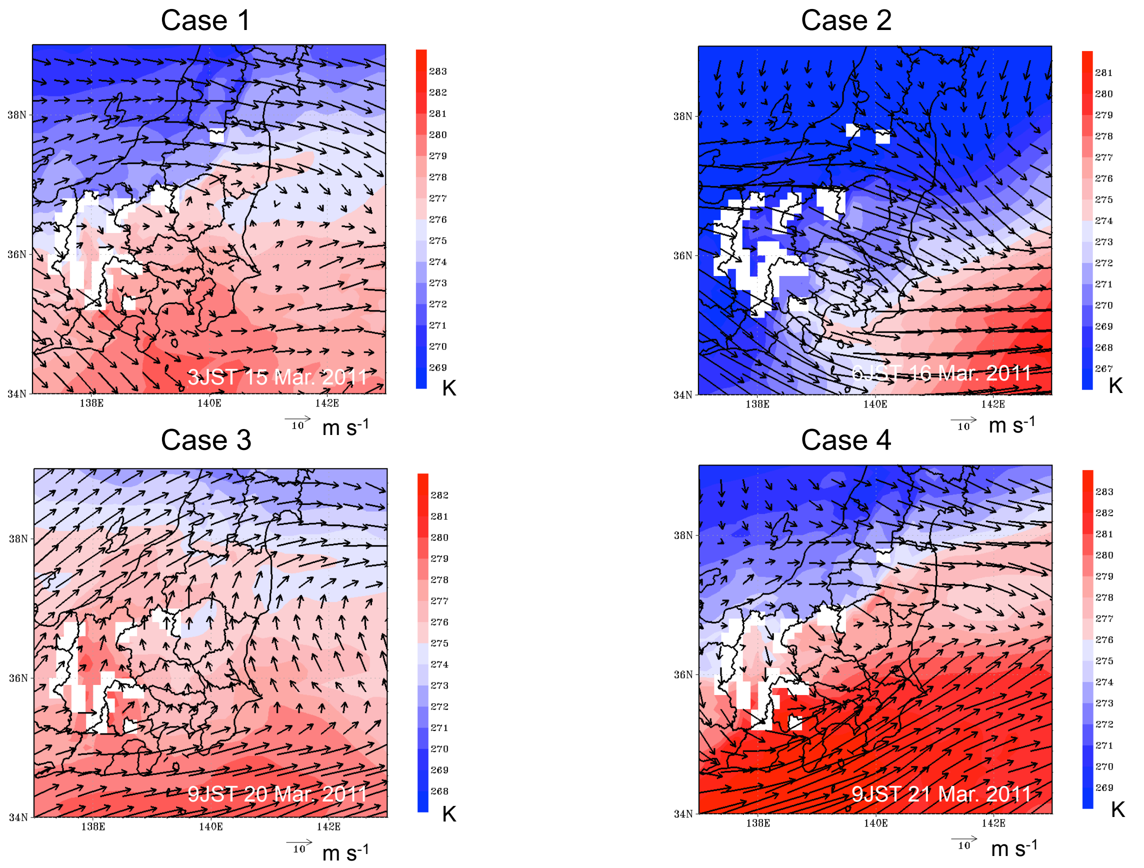

Supplementary Figure S2: **Upper airflows.** The figure is the same as the Extended Data Fig. 1 except for 850hPa. The maps were created by using GrADS 2.0.1 (http://cola.gmu.edu/grads/).

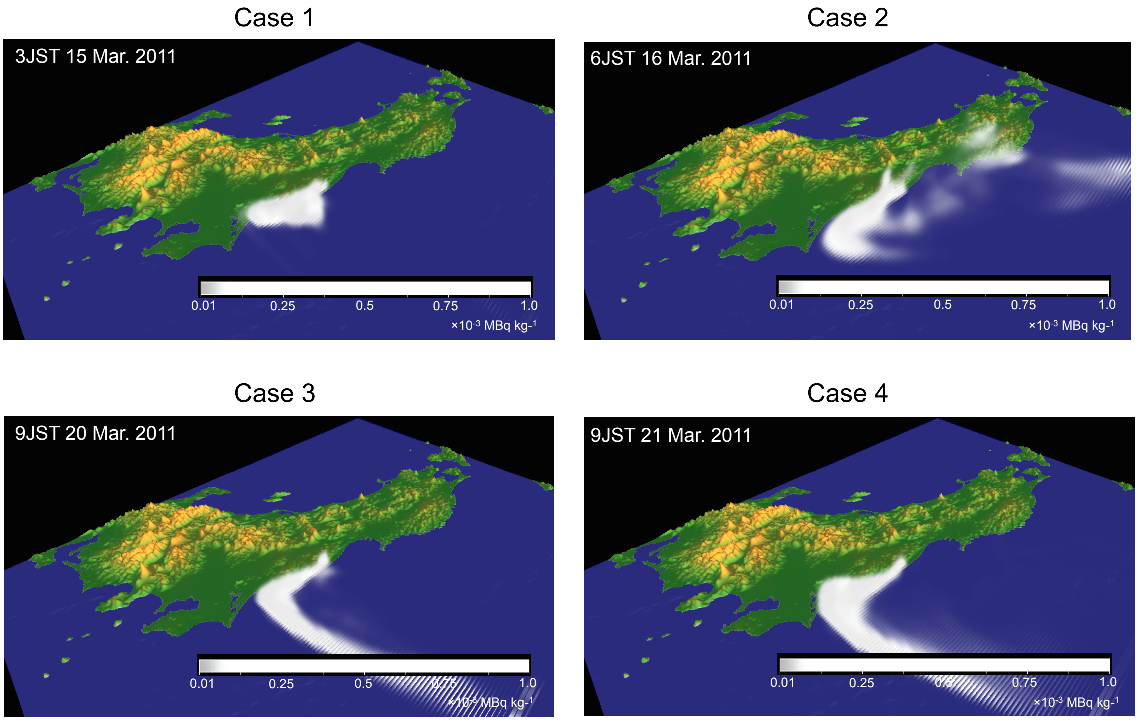

Supplementary Figure S3: **3D image of the simulated radioactive plume.** The simulated mixing ratio of ^{131}I in the cases when the high air dose observed in Tokai-mura (Fig. 1b). The maps were created by using Volume Data Visualizer for Google Earth (VDVGE) 1.1.7 ESC JAMSTEC (https://www.jamstec.go.jp/esc/research/Perception/vdvge.ja.html).

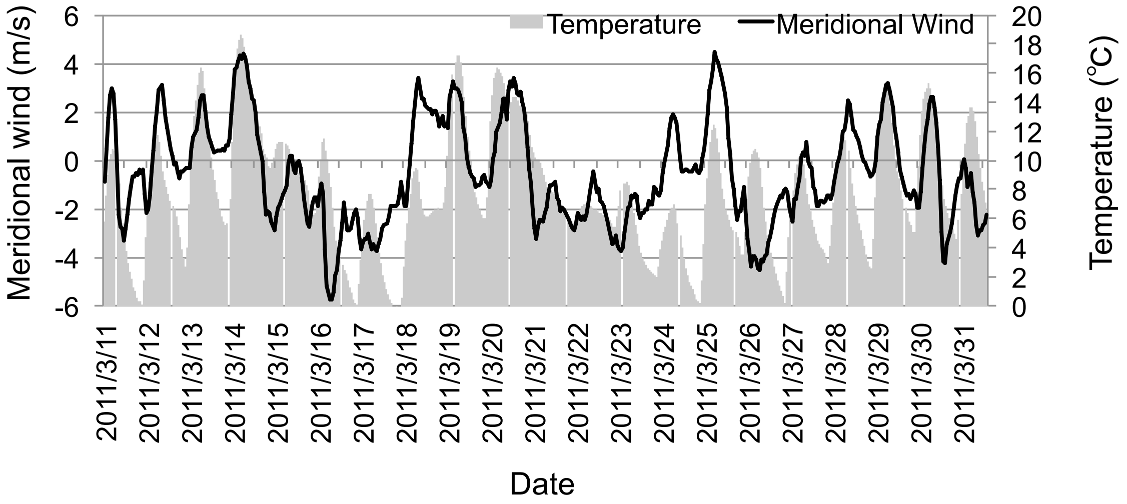

Supplementary Figure S4: **Diurnal variations in the Tokyo metropolitan area.** Time variations of the area-averaged temperature and meridional wind of station data (AMeDAS) from Mar. 11 to 31, 2011. The map was created by using Microsoft Excel for Mac 2011.

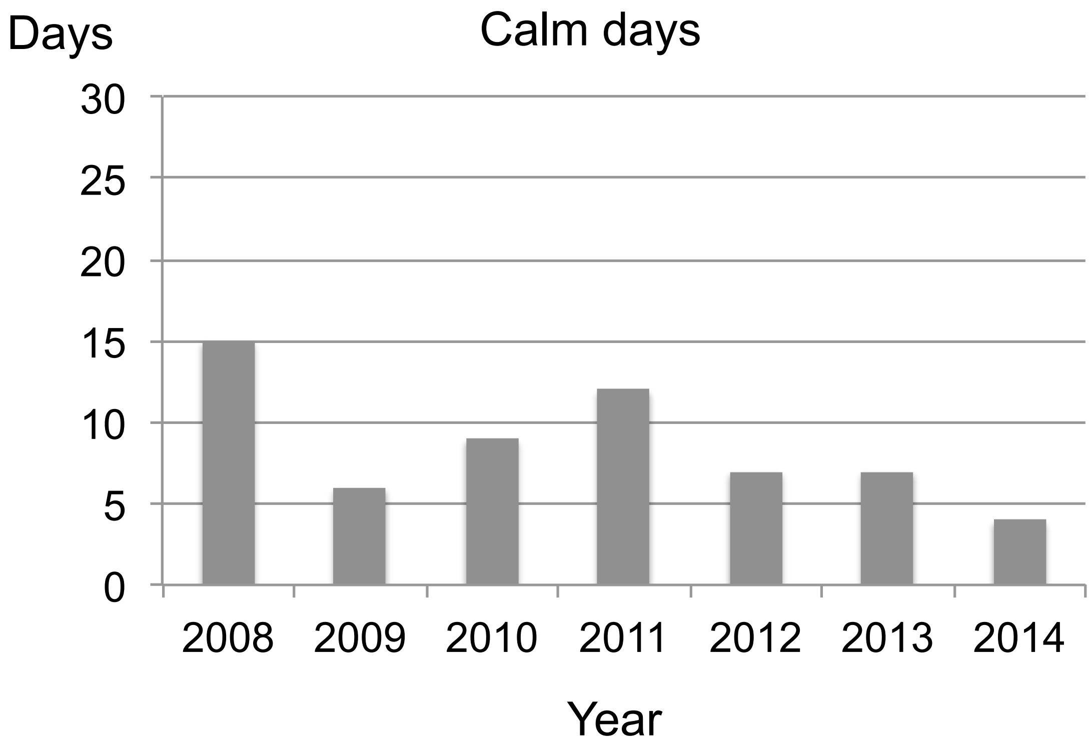

Supplementary Figure S5: **The climatic influence of the calm day.** The number of the calm days in March from 2008 to 2014. The graph was created by using Microsoft Excel for Mac 2011.

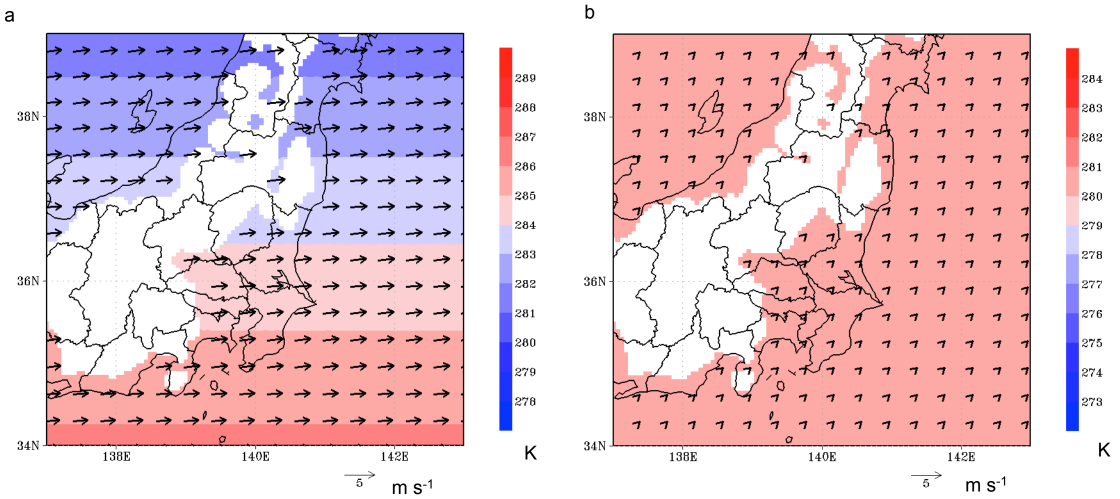

Supplementary Figure S6: **The initial conditions of Ex.1 and Ex.2.** The initial atmospheric fields of wind and temperature at 975hPa for sensitivity test. The maps were created by using GrADS 2.0.1 (http://cola.gmu.edu/grads/).

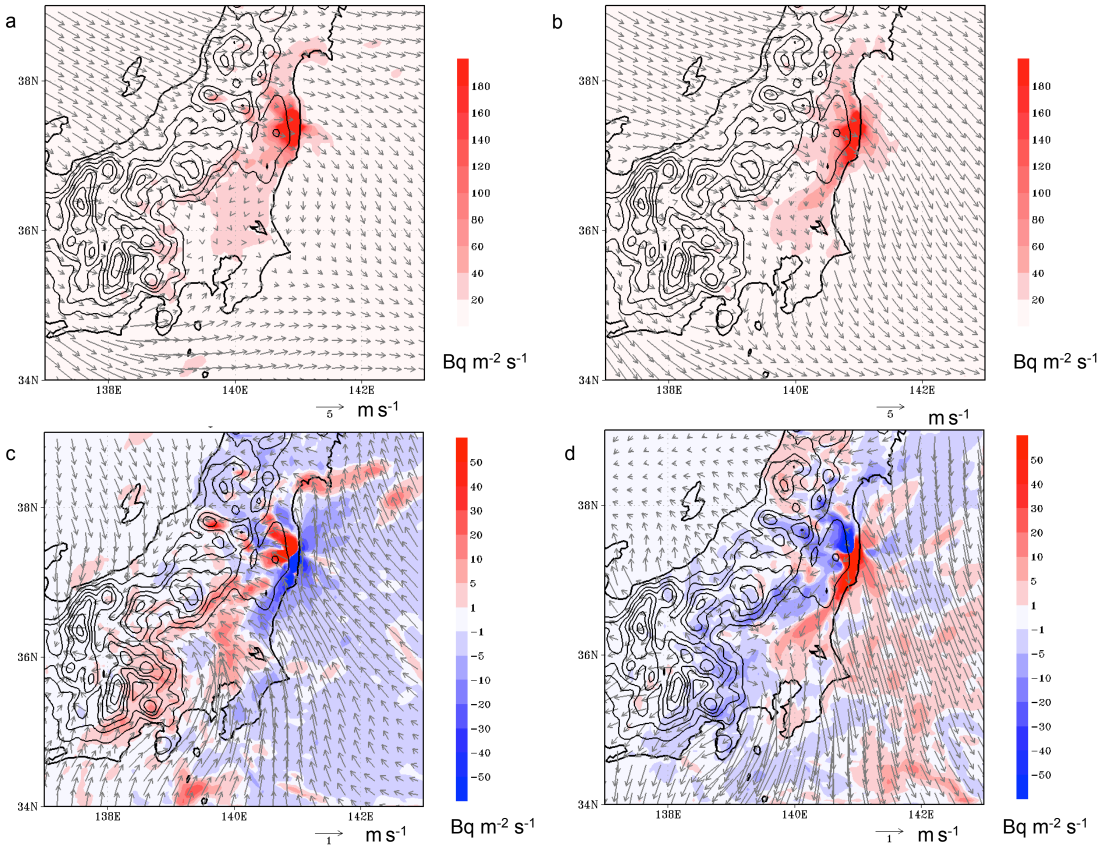

Supplementary Figure S7: **Simulated diurnal variations of radioactive material deposition.** The absolute values (upper figures) and the deviations from daily average (lower figures) of the wet and the dry depositions of ^{131}I at 6 JST and 18 JST in March from 2008 to 2014. The maps were created by using GrADS 2.0.1 (http://cola.gmu.edu/grads/).

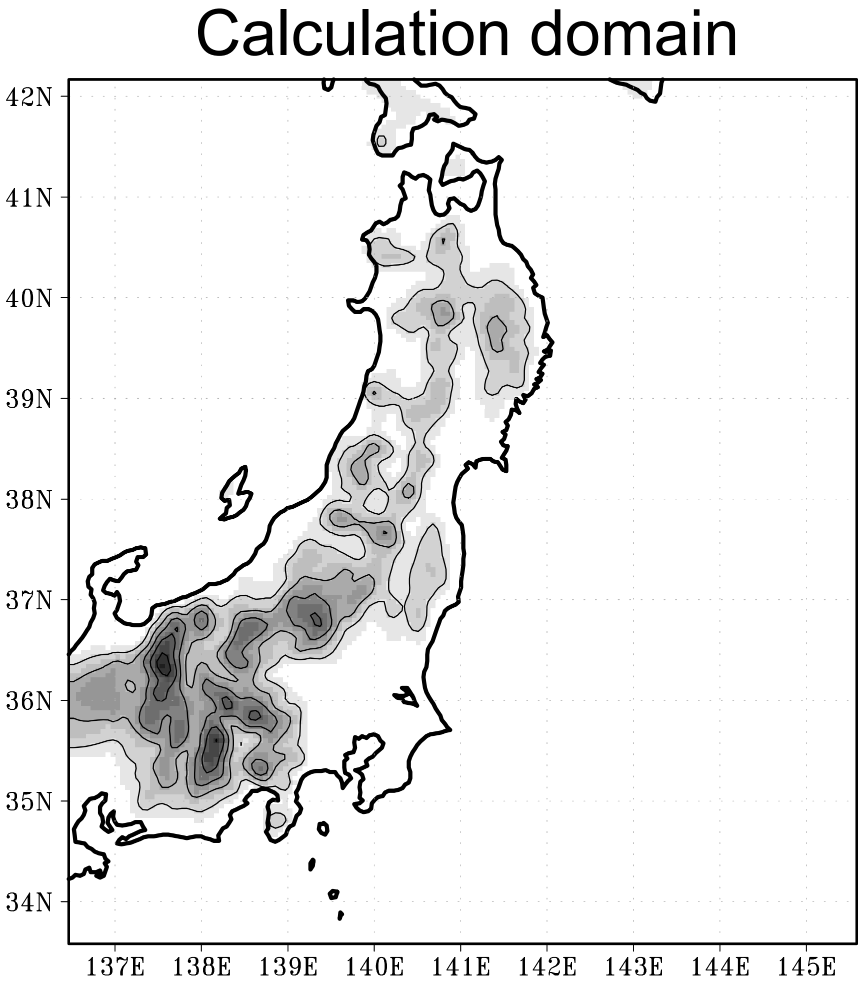

Supplementary Figure S8: **Calculation domain of hind cast and sensitivity test.** The map was created by using GrADS 2.0.1 (http://cola.gmu.edu/grads/).

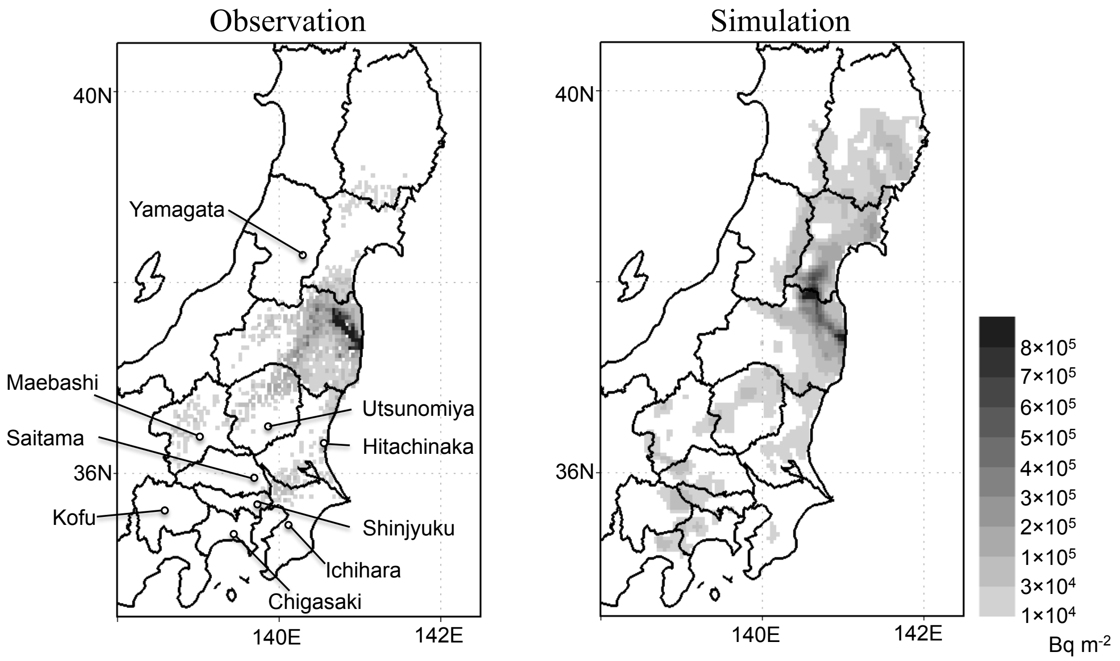

Supplementary Figure S9: **Deposition distributions of ^{137}Cs by the observation and the simulation.** The observation data is provided by MEXT. The simulated distribution map is produced using the time-integrated values from 11 to 31 Mar. 2011 in the HC simulation. The small circle shows the location of observation sites of daily fallout of ^{131}I and ^{137}Cs, which is shown in Fugures S11 and S12. The maps were created by using GrADS 2.0.1 (http://cola.gmu.edu/grads/).

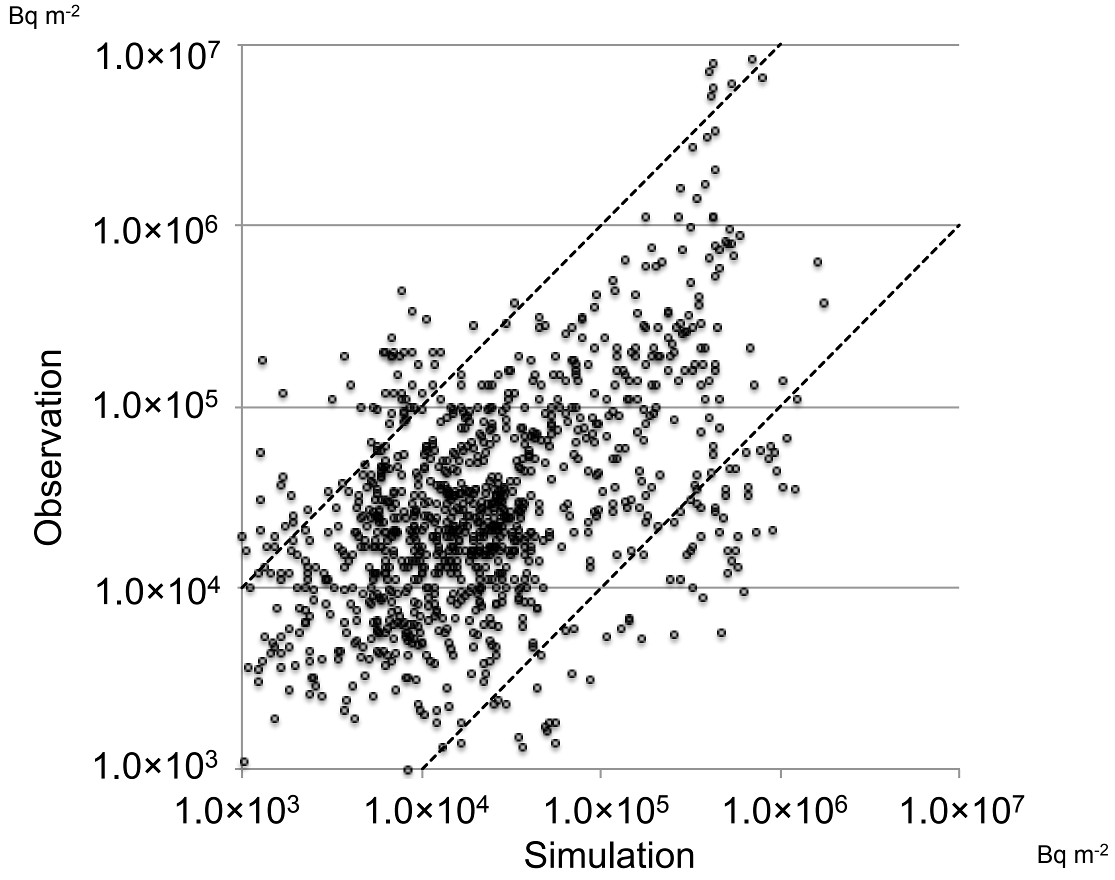

Supplementary Figure S10: **The relationship of deposition of ^{137}Cs between the observation and the simulation.** The nearest grid point to the observation site is used for validation. The graph was created by using Microsoft Excel for Mac 2011.

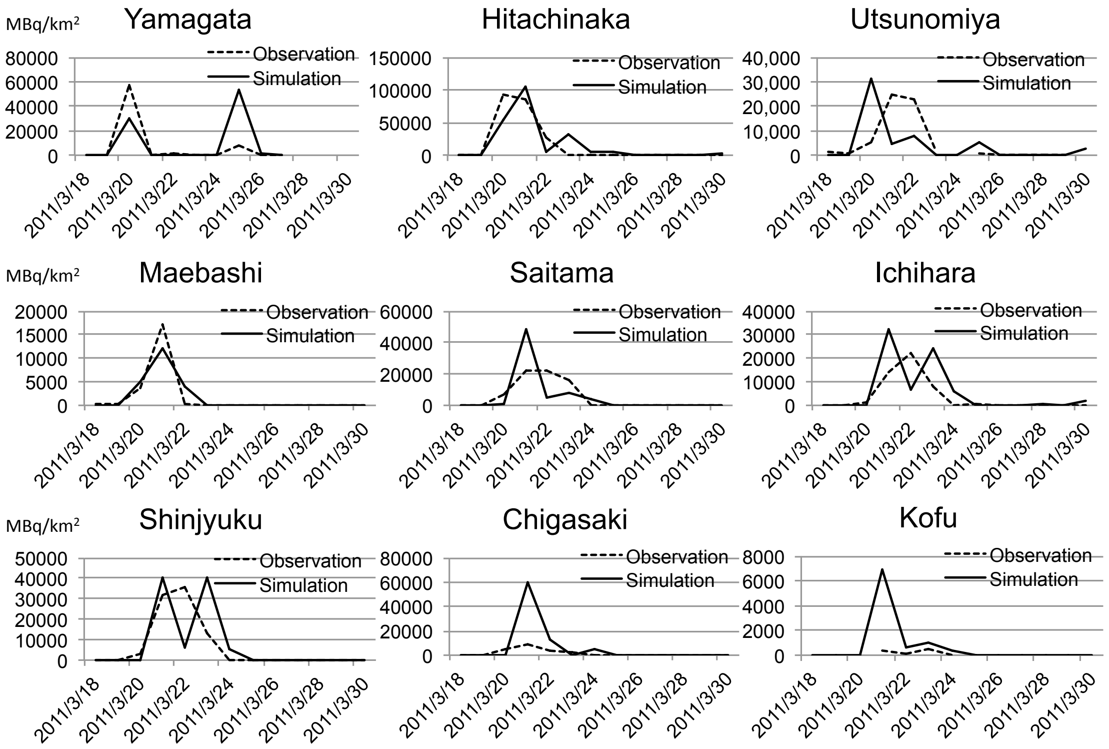

Supplementary Figure S11: **Time variation of daily deposition (fallout) of ^{131}I at the observation sites.** The graphs were created by using Microsoft Excel for Mac 2011.

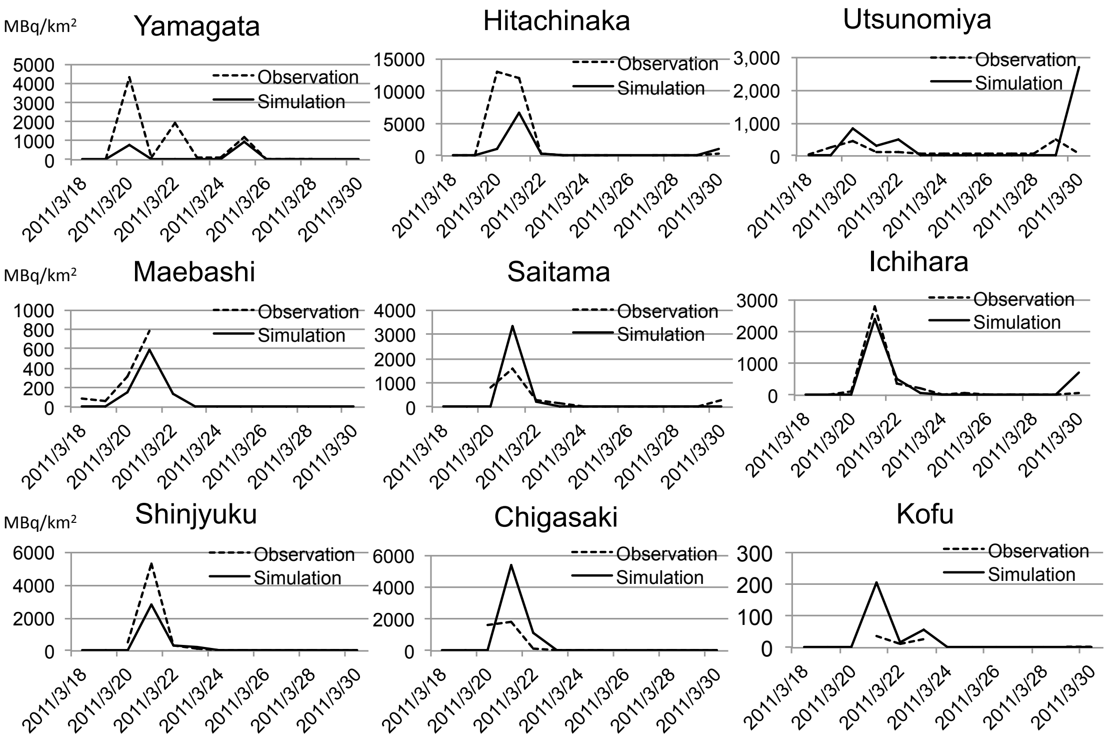

Supplementary Figure S12: **Time variation of daily deposition (fallout) of ^{137}Cs at the observation sites.** The graphs were created by using Microsoft Excel for Mac 2011.
